# Association between proximity to COVID-19 and the quality of life of healthcare workers

**DOI:** 10.1371/journal.pone.0283424

**Published:** 2023-03-23

**Authors:** Nang Ei Ei Khaing, Claire Quah, Gek Kheng Png, Joanna Wong, Augustine Tee, Hong Choon Oh

**Affiliations:** 1 Health Services Research, Changi General Hospital, Singapore, Singapore; 2 General Medicine, Changi General Hospital, Singapore, Singapore; 3 Nursing, Changi General Hospital, Singapore, Singapore; 4 Allied Health, Changi General Hospital, Singapore, Singapore; 5 Respiratory & Critical Care Medicine, Changi General Hospital, Singapore, Singapore; 6 Centre for Population Health Research and Implementation, SingHealth Office of Regional Health, Singapore, Singapore; 7 Health Services and Systems Research, Duke-NUS Medical School, Singapore, Singapore; St John’s University, UNITED STATES

## Abstract

**Background:**

The coronavirus disease 2019 (COVID-19) affects almost all countries in the world and it impacts every aspect of people’s life-physically, mentally, and socio-economically. There are several research studies examining the impact of this pandemic on health, however, very few studies examining the impact of this pandemic on quality of life. This study aimed to investigate the association between proximity to the COVID-19 and quality of life of healthcare workers and identify factors influencing quality of life.

**Methods:**

A cross-sectional study was conducted among hospital staff in a tertiary hospital in Singapore. Data on demographic, medical history, lifestyle factors, psychosocial factors, and quality of life were collected using online self-administered questionnaire. Quality of life (QoL) was measured by the WHOQOL-BREF questionnaire. Robust linear regression was used to determine factors associated with quality of life.

**Results:**

A total of 1911 participants were included in the analysis. The average age of participants was 38.25 (SD = 11.28) years old. 26.90% of participants had been quarantined, hospitalised, being suspected or diagnosed of having COVID-19 infection and they were found to have the lowest levels of QoL across all four domains (physical, psychological, social, and environmental domains). Participants who were singles or nurses, worked in shifts or worked longer hours, had chronic diseases were likely to have lower QoL scores compared to participants in other categories. Healthy lifestyle, social connectivity, resilience, social and workplace support were associated with higher QoL scores.

**Conclusions:**

In planning of measures which aim to improve QoL of healthcare workers, priority should be given to individuals who have been quarantined, hospitalised, being suspected, or diagnosed of having COVID-19 infection. In addition to the proximity of the COVID, lifestyle and psychosocial factors contribute to QoL of healthcare workers. Hence, multifaceted interventions are needed to improve QoL of healthcare workers.

## Introduction

Since the SARS-CoV-2 virus has been first identified in December 2019, more than 661 million COVID-19 cases and 6.70 million deaths have been reported globally as of 13 th January 2023 [1]. In Singapore, the first COVID-19 case was reported on January 23^rd^, 2020 [2]. Due to the rising rate in infection, Singapore imposed a national lockdown which was called as a circuit breaker from April 7^th^ to June 1^st^, 2020 [2]. After that, the infection rate was gradually declined until it was spiked again near the end of 2021 and occasional spikes in 2022. Singapore was able to maintain relatively low infection rate and fatality rate with 2,210,633 confirmed cases and 1,717 fatalities as at 13^th^ January 2023 [1].

The unprecedented scale of this pandemic affects every aspect of people’s life-physically, mentally, and socio-economically. In a study done in Taiwan, greater fear of COVID-19 was found to be significantly associated with suicidal thoughts among outpatients, healthcare workers (HCWs), and general population [3] and this finding has been supported by a recent systematic review and meta-analysis [4]. Four in every ten individuals reported a sleep problem during pandemic and the sleep disturbances was higher during lockdown [5] and sleep problems are found to be associated with psychological distress among COVID-19 patients, HCWs, and general population [6]. The prevalence of sleep problems among COVID-19 patients and HCWs was higher than that of general population [5, 6]. It may be due to that COVID-19 patients and HCWs were more likely to have experienced social stigma and discrimination [7, 8]. It has also been reported that the prevalence of psychological problems was higher among HCWs than the general population [4, 9]. High level of perceived stigma and burnout were observed among HCWs [10] and it may contribute to the higher prevalence of psychological morbidities among HCWs. The psychological impact of the COVID-19 on HCWs were consistently reported across countries globally. In study done among HCWs in India, Pakistan, and Nepal, 57% reported poor sleep quality and 10% reported psychological distress [11]. Similar findings were observed in other populations. A significant proportion of HCWs in Taiwan reported post-traumatic stress disorder symptoms, insomnia, and depressive symptoms [12] and HCWs in African countries reported a range of psychological problem including depression, anxiety, and insomnia [13]. Although there is ample evidence of the impact of the pandemic on health, there are very few studies examining the impact of this pandemic on the quality of life (QoL) of the population especially the QoL of HCWs. Quality of life encompasses not only physical and mental health but also personal beliefs, social relationships, and their relationship to salient features of their environment [14]. It has been reported that the QoL of the population during the pandemic was lower than the pre-pandemic level [15]. Healthcare workers have a higher risk of psychological morbidities compared to the general population [9] and it may likely be the same for QoL. It is crucial to understand the impact of the pandemic on the QoL of HCWs and risk factors associated with it as QoL of healthcare workers, directly and indirectly, affects patients’ safety and quality of care [16].

In a recent reported review on QoL assessment of healthcare professionals during COVID-19 where 23 studies were reviewed [17], only nine studies assessed the overall QoL of HCWs [18–26] and majority of the studies only assessed psychological health or health related QoL [17]. Of these nine studies, only three studies assessed the risk factors for QoL [19, 24, 25]. Abdelghani et al conducted a cross-sectional study among 218 HCWs and found that health anxiety to COVID-19 virus was inversely correlated with all domains of QoL [25]. A study conducted among 240 healthcare professionals in Turkey found that stress, trait anxiety, and burnout were significantly associated with QoL [24]. In a study of Suryavanshi et al, moderate and severe anxiety and depression were significantly associated with QoL among 197 healthcare professionals [19]. In addition to studies reported in the review, there were two studies assessing risk factors for QoL among HCWs [27, 28]. Rashid et al reported socio-demographic risk factors associated with QoL among 322 HCWs from Bangladesh [28]. In a study done by Woon et al among 389 university-based HCWs, it was found that COVID-19-related stressors and psychological sequelae were associated with lower QoL and perceived social support associated with higher QoL [27]. With the exception of the study done by Woon et al who have evaluated the impact of psychosocial factors on QoL, all the remaining aforementioned papers did not study the effect of proximity to COVID-19, lifestyle and psychosocial factors on QoL.

This study aims to address current research gap by examining the QoL of HCWs in relation to the proximity to COVID-19 and identifying socio-demographic, lifestyle, and psychosocial factors influencing QoL of HCWs during pandemic. We hypothesize that participants with higher level of proximity to the COVID-19 would have lower QoL than participants with no proximity to the COVID-19.

## Materials and methods

### Study design and study population

This cross-sectional study was conducted among HCWs in a tertiary hospital from mid-December 2020 to the end of January 2021. An email containing an invitation link to the online survey was distributed through hospital’s mass communication system. A total of 1919 HCWs participated in the study and the response rate was 34.13%.

During this study period, Singapore’s Disease Outbreak Response System Condition (Dorscon) was under the “orange” level where COVID-19 was considered a severe disease which could spread easily from person to person and restrictions were imposed to contain the spread of disease [2, 29]. These restrictions included group size limit of five persons outside residential properties, and every household could receive up to five visitors at any time (this limit was raised to eight on 28 Dec 2020) [30]. The COVID-19 vaccination for healthcare staff in the participating institution was commenced on 8 January 2021 [31].

### Data collection

Data on sociodemographic, medical history, lifestyle factors, perceived social and organizational support, personal resilience, and proximity to the COVID-19 were collected with an online self-administered questionnaire in the English language through a secured web-based platform, Qualtrics.

Proximity to the COVID-19 was assessed by asking “Have you experienced the following since the start of the pandemic?” with “yes” and “no” options for 10 items on the history of COVID-19 infection or in close contact with COVID-19 positive cases at work or personal level. In these 10 items, participants were asked whether or not they have family member/s, friends, co-workers, someone living with them diagnosed with COVID-19 infection, have been assigned to work in COVID-19 isolation facilities/wards or have been in any contact or close proximity with COVID-19 patients, have been quarantined or hospitalised, or being suspected or diagnosed of having COVID-19 infection. We categorised the proximity into five levels; level 0 was no proximity (i.e no to all 10 questions), level 1 was having friends and co-workers being diagnosed with COVID-19 infection, level 2 was having co-habitants, and family members being diagnosed with COVID-19 infection, level 3 was assigned to work in COVID-19 isolation facilities/wards or having been in any contact or close proximity with COVID-19 patients, and level 4 was having been quarantined or hospitalised, or being suspected or diagnosed of having COVID-19 infection. If participants have more than one possible level, they are categorized in the highest level as we assume that the effect of the highest level would be dominant over other possible levels. For example, if participants had friends with COVID-19 (Level 1) and been also assigned to work in COVID-19 isolation (Level 3), these participants were be categorized in the higher level (i.e. Level 3).

Quality of life (QoL) was measured by the WHOQOL-BREF questionnaire which is shown to have excellent psychometric properties of reliability [14]. It contains 26 questions assessing QoL in four domains: physical, psychological, social relationships, and environment. Data cleaning, checking, scores computing, and transforming were done according to WHOQOL-BREF instructions, resulted in a 0–100 scale [32]. A higher score indicated a higher level of QoL.

Perceived social support was measured by the F-SozU K-6 which is well-validated and designed to measure social support and included six questions, asking participants to rate perceived or anticipated social support in a five-point Likert scale with a total score range of 6 to 30. A higher score indicated a higher level of perceived social support [33]. Resilience was measured brief resilience scale (BRS) which is a reliable instrument to assess to the ability to bounce back or recover from stress [34]. It contained six questions asking participants to rate each item from strongly disagree to strongly agree and a possible total score ranged from 6 to 30. A higher score indicated a higher level of resilience. Perceived organizational support (POS) was measured by the 8-item Survey of Perceived Organizational Support (SPOS) developed by Eisenberger et al that is based on organizational support theory [35]. As previously done by Eisenberger et al and others [36, 37], we adopted five high loading items (#4, #8, #9, #23, and #25) and two high loading items (#8 and #9) from the 36-item SPOS to assess perceived supervisor support (PSS) and perceived peer support (PPS) respectively. Participants rated their agreement to each statement with options ranging from 0 = “strongly disagree” to 6 = “strong agree”. The score for workplace support was obtained by summing up the scores from organizational support, supervisor support, and peer support and a possible total score from 15 items ranged from 0 to 90. A higher score indicated a higher level of organizational support.

Lifestyle factors (sufficient sleep, nutritious diet, exercise, smoking, and alcohol intake), spiritual activities (praying), and social connectivity (talking to families or friends in-person/online/on the phone) were assessed by asking participants how often they did these activities in the past week, with five options ranging from “none of the time” to “all of the time” and scores assigned from 1 to 5 respectively. For smoking and alcohol intake, the responses were coded reversely. The lifestyle index was calculated by combing the scores from these five items, with a possible total score ranged from 5 to 25. A higher score indicated a healthier lifestyle.

### Ethic statement

Ethics approval was obtained from the SingHealth institutional review board (CIRB Ref: 2020/3038). Implied consent was assumed once participants clicked on the “next” button to proceed to the questionnaire after reading the study information on the first page.

### Statistical analysis

Eight participants with more than 20% of missing QoL data were excluded from analysis according to the WHO-BREF guideline [32]. A total of 1911 participants were included in the analysis. Means and standard deviations were calculated for normally distributed variables and medians and inter-quartile ranges were summarized for skewed variables. The independent t-test and the analysis of variance method were used to compare means of continuous variables between the groups. Multivariate linear regressions were used to examine factors associated with QoL and robust estimates of variance were obtained.

All statistical tests were two sided with the level of significance defined as p-value < 0.05.

## Results

The average age of participants was 38.25 (SD = 11.28) years old. The majority of participants were female (88.70%), local (76.61%), nurses (62.06%), and on rotating shift/permanent shift (61.73%). 11.44% have a chronic disease and 26.90% had been quarantined, hospitalised, being suspected or diagnosed of having COVID-19 infection (Table 1).

**Table 1:** Characteristics of the study population.

Age (mean±SD)	38.25±11.28
Gender (N, %)	
Male	216 (11.30)
Female	1695 (88.70)
Ethnicity (N, %)	
Chinese	853 (44.78)
Malay	430 (22.57)
Indian	198 (10.39)
Others	424 (22.26)
Marital status (N, %)	
Never married	770 (40.40)
Currently married	1045 (54.83)
Separated but not divorced/divorced/widowed	91 (4.77)
Resident status (N, %)	
Local	1464 (76.61)
Non-local	447 (23.39)
Job category (N, %)	
Administrative staff/ Operation staff/other*	163 (8.53)
Doctors	31 (1.62)
Nurses	1186 (62.06)
Allied Health Professionals	405 (21.19)
Ancillary and support staff	126 (6.59)
Total work hours per week (mean±SD)	43.55±4.94
Year of experiences in current profession (N, %)	
<3 year	399 (21.14)
3.1–5 year	195 (10.33)
5.1–10 year	546 (28.93)
>10 year	747 (39.59)
Work Schedule (N, %)	
Non-shift	720 (37.70)
Shift	1179 (61.73)
Part-time	11 (0.58)
Have chronic disease (N, %)	218 (11.44)
Proximity to Covid (N,%)	
Level 0	889 (49.61)
Level 1	64 (3.57)
Level 2	20 (1.12)
Level 3	337 (18.81)
Level 4	482 (26.90)
Have religion (N, %)	1641 (85.92)
Lifestyle index (mean±SD)	18.43±2.24
Spiritual activities (mean±SD)	2.95±1.38
Social connectivity (mean±SD)	3.54±1.00
Workplace support (mean±SD)	55.69±15.94
Social support (mean±SD)	21.47±4.35
Resilience (mean±SD)	19.62±3.51

*other-tele carer, Central sterile supplies department technician, community care, renal dialysis unit, community coordinator, research coordinators

Participants in the level 4 proximity to the COVID-19 were found to have the lowest levels of QoL across all four domains. Nursing staff has the lowest level of QoL in physical and environmental domains while allied health professionals have the lowest QoL in psychological and social domains. Participants with more than 10 years of professional experience or on non-shift work schedules have a higher level of QoL across all four domains. (Table 2).

**Table 2:** Factors associated with QoL (univariate analysis).

	Physical		Psychological		Social		Environment	
	mean±SD	p value	mean±SD	p value	mean±SD	p value	mean±SD	p value
Proximity to Covid								
Level 0	66.20±13.76	<0.001	62.17±14.27	<0.001	64.70±15.66	<0.01	62.97±13.00	<0.001
Level 1	65.12±14.73		59.90±14.39		63.62±17.08		60.50±13.89	
Level 2	62.29±13.92		62.50±10.81		70.00±12.80		61.41±11.07	
Level 3	64.53±15.29		60.98±15.49		63.53±16.71		62.06±13.73	
Level 4	60.71±13.54		57.66±14.58		61.23±16.56		59.27±12.58	
Gender								
Male	66.12±14.93	0.06	60.28±16.02	0.67	61.03±17.56	0.01	61.80±15.05	0.93
Female	64.21±14.11		60.74±14.50		64.01±16.10		61.71±12.89	
Ethnicity								
Chinese	65.43±14.43	<0.001	58.89±14.97	<0.001	61.95±15.81	<0.001	61.29±13.83	0.22
Malay	62.14±14.01		60.66±14.38		64.95±16.63		61.49±13.18	
Indian	63.89±13.32		61.09±14.78		63.97±18.41		61.60±12.00	
Others	64.99±14.11		64.15±13.74		65.81±15.43		62.89±12.12	
Marital status								
Never married	62.45±13.89	<0.001	57.51±14.92	<0.001	59.35±14.99	<0.001	60.34±13.45	<0.001
Currently married	65.64±14.24		62.88±13.93		67.38±15.73		62.65±12.74	
Separated /divorced/widowed	67.17±14.41		62.41±16.06		57.55±21.61		62.71±14.06	
Resident status								
Local	64.49±14.41	0.71	60.09±14.84	<0.001	63.68±16.60	0.96	61.78±13.53	0.71
Non-local	64.21±13.56		62.65±13.95		63.64±15.30		61.52±11.83	
Religion								
No	64.00±13.75	0.59	57.22±14.68	<0.001	60.22±16.12	<0.001	59.48±13.68	0.00
Yes	64.50±14.29		61.26±14.60		64.26±16.24		62.09±13.03	
Job category								
Administrative staff/ Operation staff/other*	69.06±14.16	<0.001	62.60±14.06	0.02	63.96±16.81	0.04	66.06±13.70	<0.001
Doctors	76.38±14.83		64.65±17.51		66.94±19.42		68.35±16.05	
Nurses	62.71±14.14		60.52±14.64		63.94±16.11		60.73±12.94	
Allied Health Professionals	66.27±13.40		59.39±14.77		61.75±15.92		62.30±12.77	
Ancillary and support staff	65.67±13.95		62.94±14.26		66.17±17.35		61.96±13.35	
Year of experiences in current profession								
<3 year	62.75±13.14	<0.001	58.10±14.41	<0.001	62.20±16.27	<0.001	60.60±13.39	<0.001
3.1–5 year	62.25±15.47		58.20±14.52		62.99±15.01		59.00±14.23	
5.1–10 year	62.98±14.10		59.36±15.26		61.94±17.31		60.74±12.80	
>10 year	67.04±14.22		63.70±14.00		66.05±15.55		63.73±12.83	
Work Schedule								
Non-shift	67.63±13.76	<0.001	62.03±13.61	0.00	64.17±16.33	0.05	64.13±13.06	<0.001
Shift	62.42±14.15		59.82±15.25		63.30±16.28		60.19±13.02	
Part-time	70.13±10.14		66.29±12.00		74.24±5.84		68.63±5.50	
Have chronic disease								
Yes	63.17±15.44	0.16	60.57±15.07	0.89	62.04±16.84	0.12	61.96±14.30	0.78
No	64.60±14.05		60.71±14.63		63.89±16.18		61.70±13.00	

*other-tele carer, Central sterile supplies department technician, community care, renal dialysis unit, community coordinator, research coordinators

In multivariate models, compared to the participants in level 0 (no proximity), participants in the level 4 proximity to COVID-19 have a significantly lower level of QoL in all domains except environmental domains. Nursing staff has a lower level of physical and environmental QoL while doctors have a higher level of physical QoL compared to administrative and operation staff. Ancillary and support staff also has a lower level of QoL in the environmental domain. Healthy lifestyle and psychosocial factors (social connectivity, social and workplace support, and resilience) are consistently associated with a higher level of QoL in all four domains. In addition, spiritual activities were also significantly associated with better psychological QoL (Table 3a & 3b).

**Table 3:** Factors associated with quality of life (multivariate analysis).

(a)						
	Physical Domain			Psychological domain		
	Coefficient	Standardized coefficient	p value	Coefficient	Standardized coefficient	p value
Proximity to Covid						
Level 0	Reference			Reference		
Level 1	0.82	0.01	0.57	0.35	0.005	0.81
Level 2	-2.87	-0.02	0.22	-1.31	-0.01	0.51
Level 3	-0.68	-0.02	0.37	-0.66	-0.02	0.39
Level 4	-2.63	-0.08	<0.001	-1.80	-0.05	0.01
Age	0.06	0.04	0.14	0.05	0.03	0.22
Gender						
Male	Reference			Reference		
Female	-1.40	-0.03	0.10	-0.84	-0.02	0.36
Ethnicity						
Chinese	Reference			Reference		
Malay	-0.77	-0.02	0.39	1.15	0.03	0.21
Indian	-0.60	-0.01	0.58	0.07	0.00	0.95
Others	-0.50	-0.01	0.64	1.69	0.05	0.15
Marital status						
Never married	Reference			Reference		
Currently married	-0.34	-0.01	0.60	1.45	0.05	0.03
Separated /divorced/widowed	1.90	0.03	0.17	2.88	0.04	0.06
Resident status						
Local	Reference			Reference		
Non-local	1.61	0.05	0.08	2.13	0.06	0.04
Religion						
No	Reference			Reference		
Yes	-0.07	0.00	0.94	-0.54	-0.01	0.56
Job category						
Administrative staff/ Operation staff/other*	Reference			Reference		
Doctors	4.55	0.04	0.02	0.21	0.00	0.92
Nurses	-3.05	-0.10	0.01	1.05	0.03	0.35
Allied Health Professionals	-0.52	-0.02	0.64	-0.16	0.00	0.89
Ancillary and support staff	-1.05	-0.02	0.46	1.28	0.02	0.42
Year of experiences in current profession						
<3 year	Reference			Reference		
3.1–5 year	-0.30	-0.01	0.78	-0.64	-0.01	0.53
5.1–10 year	0.36	0.01	0.65	-0.78	-0.02	0.37
>10 year	0.42	0.01	0.64	0.01	0.00	0.99
Work Schedule						
Non-shift	Reference			Reference		
Shift	-1.53	-0.05	0.02	-0.34	-0.01	0.62
Part-time	-3.84	-0.02	0.08	1.96	0.01	0.48
Have chronic disease						
Yes	Reference			Reference		
No	3.76	0.08	<0.001	1.69	0.04	0.08
Work hours per week	-0.12	-0.04	0.03	-0.03	-0.01	0.69
Lifestyle index	1.93	0.30	<0.001	1.87	0.28	<0.001
Spiritual activities	-0.31	-0.03	0.29	0.85	0.08	0.01
Social connectivity	0.91	0.06	0.01	0.96	0.07	0.01
Workplace support	0.20	0.22	<0.001	0.14	0.15	<0.001
Social support	0.48	0.14	<0.001	0.60	0.17	<0.001
Resilience	0.68	0.17	<0.001	0.99	0.24	<0.001
(b)						
	Social domain			Environmental domain		
	Coefficient	Standardized coefficient	p value	Coefficient	Standardized coefficient	p value
Proximity to Covid						
Level 0	Reference			Reference		
Level 1	1.34	0.02	0.48	0.04	0.00	0.98
Level 2	3.54	0.03	0.18	-0.94	-0.01	0.69
Level 3	-0.24	-0.01	0.78	-0.20	-0.01	0.77
Level 4	-1.28	-0.04	0.13	-1.18	-0.04	0.05
Age	-0.02	-0.02	0.60	0.01	0.01	0.78
Gender						
Male	Reference			Reference		
Female	1.99	0.04	0.07	-0.59	-0.01	0.50
Ethnicity						
Chinese	Reference			Reference		
Malay	1.05	0.03	0.34	0.91	0.03	0.27
Indian	0.59	0.01	0.69	0.08	0.00	0.94
Others	1.81	0.05	0.17	1.41	0.05	0.16
Marital status						
Never married	Reference			Reference		
Currently married	5.78	0.18	<0.001	-0.91	-0.03	0.13
Separated /divorced/widowed	-1.06	-0.01	0.60	-0.50	-0.01	0.71
Resident status						
Local	Reference			Reference		
Non-local	-2.63	-0.07	0.02	-0.71	-0.02	0.42
Religion						
No	Reference			Reference		
Yes	-0.14	0.00	0.90	0.61	0.02	0.49
Job category						
Administrative staff/ Operation staff/other*	Reference			Reference		
Doctors	3.21	0.03	0.26	0.04	0.00	0.98
Nurses	0.85	0.03	0.58	-3.39	-0.12	<0.001
Allied Health Professionals	-0.03	0.00	0.99	-1.79	-0.06	0.12
Ancillary and support staff	3.26	0.05	0.08	-3.64	-0.06	0.02
Year of experiences in current profession						
<3 year	Reference			Reference		
3.1–5 year	-0.54	-0.01	0.67	-1.26	-0.03	0.20
5.1–10 year	-2.55	-0.07	0.02	0.15	0.01	0.85
>10 year	-0.84	-0.03	0.48	0.98	0.04	0.26
Work Schedule						
Non-shift	Reference			Reference		
Shift	1.60	0.05	0.07	-1.51	-0.06	0.02
Part-time	6.64	0.03	<0.001	1.31	0.01	0.47
Have chronic disease						
Yes	Reference			Reference		
No	1.86	0.04	0.08	0.66	0.02	0.46
Work hours per week	0.03	0.01	0.67	-0.06	-0.02	0.28
Lifestyle index	1.11	0.15	<0.001	1.49	0.25	<0.001
Spiritual activities	0.43	0.04	0.24	0.02	0.00	0.93
Social connectivity	2.16	0.14	<0.001	1.23	0.09	<0.001
Workplace support	0.07	0.07	0.02	0.18	0.21	<0.001
Social support	1.23	0.33	<0.001	0.92	0.30	<0.001
Resilience	0.38	0.09	<0.001	0.36	0.10	<0.001

## Discussion

In this study, we found that participants who have been quarantined, hospitalised, being suspected, or diagnosed of having COVID-19 infection have lower levels of physical, psychological, and environmental QoL compared to participants with no proximity to the COVID-19. This shows that proximity to the COVID-19 is associated with various aspects of HCWs’ lives. In previous studies, it has been reported that quarantined HCWs were more likely to suffer from acute stress disorders, poor concentration and indecisiveness, deteriorating work performance, and reluctance to work or consideration of resignation [38], and those quarantined for more than 10 days showed significantly higher post-traumatic stress symptoms than those quarantined for less than 10 days [39].

We did not observe significant difference in social QoL among different levels of proximity to COVID-19. It may be that regardless of their exposure status, all participants were similarly affected by nationwide social restriction measures imposed by the government.

Nursing staff was found to have a lower level of QoL in physical and environmental domains compared to administrative and operation staff. It may be that nursing staff has higher exposure to the COVID-19 as they spend more time in the wards and provide direct care to patients [40]. This could also contribute to the observation that nurses assigned to in-patient wards have lower level of QoL than those assigned to out-patient clinics (61.31 vs 67.47 for physical domain, 59.72 vs 64.15 for psychological domain, 64.23 vs 65.98 for social domain, and 58.77 vs 63.63 environmental domain respectively). Previous studies have shown that nurses have higher rates of anxiety and depression compared to doctors [40] and these psychological factors were predictors of lower QoL among HCWs [27]. These observations might be attributed to majority of nursing staff being deployed in shifts as it was noted in our study that staff who worked in shifts have lower physical and environmental QoL than those who did not work in shifts. The effect of shift work on health is well-established. Shift work has been shown to be negatively associated with physical and mental health, QoL, and organizational outcomes such as performance and safety [41, 42].

The risk profile of participants in this study was similar to the one identified in another study conducted among HCWs in Malaysia [27]. Participants who had at least one chronic disease, worked longer hours, and were singles tends to have lower QoL, while participants with higher social support had better QoL [27]. In addition, non-local participants in our study were found to have higher psychological QoL but lower social QoL compared to local participants. The migrant population tends to have a higher level of resilience which may explain a higher level of psychological QoL [43]. However, in our study, we did not observe a significant difference in resilience levels between local and non-local participants. It may be that BRS is not able to capture all aspects of resilience as resilience is multidimensional. For the social QoL, the social network of non-locals may be smaller than locals which might affect their social QoL especially during the pandemic where restriction of social gathering or house visit is imposed, social gathering at public places or residential homes was limited, and social gathering at workplaces was prohibited. These restrictions further limited the opportunity for non-locals to expand or even maintain their social networks.

There are five protective factors emerging from this study that are independently associated with higher Qol in all domains, namely healthy lifestyle, social connectivity, resilience, social and workplace support. The beneficial effect of exercise, balanced nutrition, and adequate sleep are well established, and the health benefits of these lifestyle factors are still effective in the face of pandemics [44–46]. The protective effect of resilience, social support, and workplace support on mental health has also been reported in previous studies [47, 48]. Resilience enables people to quickly recover from adversities and social and workplace supports act as a buffer against stress, attenuates fear and uncertainty, and reduce the effect of physiological and psychological threats [47, 49]. Perceived organizational support is also shown to be associated with employee performance and well-being [35]. Social connectivity is a way to reach to these resources for coping with adversities and an important predictor for health and well-being [50]. However, the unique nature of this pandemic which requires social distancing to reduce infection hinders people to acquire the resources that they need to cope with this pandemic and Smith et al have termed this as the COVID-19 Connectivity Paradox [51]. They have suggested some solutions to overcome this including telephoning and virtual programs to maintain social connectivity. In a recent randomised clinical trial, it was reported that 10-minute telephone calls delivered by a trained layperson reduced loneliness, anxiety, and depression among adults during the COVID-19 pandemic [52]. It has been suggested that social connectivity and digital technologies act as a social cure during pandemic [53]. Consistent with these findings, social connectivity was significantly associated with better QoL in our study.

### Practical organizational implications

Employee well-being is a shared responsibility, and it is crucial that organizations take an active role in supporting the wellness of their employees. Perceived organizational support refers to an employee’s perception that the organization values his or her work contributions and cares about the employee’s well-being [35]. Such perceived support benefits not only employees but also the organization as it positively affects employees work engagement [54], organizational commitment [55], and organizational citizenship behaviour of the employees [56].

Based on our findings, organizations can initiate new measures that offer better support to the high-risk groups and promote protective factors among employees to improve the QoL of HCWs. For those who have been quarantined or hospitalized, the support should continue beyond the event as there is a persisting impact on the QoL of covid-19 survivors as well as their partners and family members [57]. Moreover, it has been reported that there is a long-term psychological impact of quarantine among hospital staff even after three years of the SARS outbreak [58] and organizational support reduced PTSD among HCWs in Wuhan, China during the COVID-19 pandemic [59]. This highlights the importance of continuing organizational support for this vulnerable group even after the events.

For protective factors, it is important that organizations invest in workplace health promotion programs to encourage staff to maintain and adopt healthier lifestyles as those with higher organizational support tends to participate in health promotion program [60]. Organizations could leverage on technology for workplace interventions during pandemic as it was challenging to implement face-to-face programs during the pandemic due to strict safety measures. In the study done among university employees, participants in the remotely supervised exercise program and the in-person-supervised exercise program demonstrated similar improvement in physical activity level, adherence to the Mediterranean diet, and an increase in health-related QoL (HrQoL) [61]. Hence, it is feasible to improve lifestyle during pandemic through remotely supervised programs. Intervention programs which aim to improve the resilience of employees may also be beneficial to organizations as studies have shown that workplace resilience trainings are found to be effective in reducing stress and improving resilience [62]. To improve resilience, Rieckert et al provided a series of recommendations on building the resilience of HCWs before and during the pandemic at individual, organizational, and environmental levels [63]. The recommended strategies before pandemic focus on education, training, and preparedness, and during pandemic emphasize on communication, psychosocial support and treatment, and monitoring health status of employees. Thus, building resilience is a continuous process and healthcare organizations may incorporate these strategies into regular staff wellness programs. Although this recommendation is for different phases of pandemic, organizations that did not have pre-pandemic trainings and preparedness may incorporate them into current plan for pandemic. For instance, the strategy adopted by the psychosocial pandemic committee (PPC) of Mount Sinai Hospital in Toronto includes education training sessions and communication [64].

Organization could also encourage staff for the COVID-19 and flu vaccination. In a study done in Taiwan, not having COVID-19 and flu vaccination is found to be associated with poor QoL [65]. Although the vaccine hesitancy was low among HCWs compared to the general population, there was still a considerable proportion of HCWs hesitant to take vaccines especially at the initial phase of vaccination. The prevalence of vaccine hesitancy among healthcare workers was 47% in India [66], 32% in Iran [67], 37% in Hong Kong [31] and 5.1% to 48.9% in Singapore [31, 68, 69]. It is crucial to identify factors associated with vaccine hesitancy and address them. One of the modifiable factors associated with vaccine hesitancy among Singapore HCWs was obtaining information from newspapers [68]. Koh et al suggested tailored education, risk messaging, and strategic legislation to reduce vaccine hesitancy [70]. In a study done in India, vaccine hesitancy was improved after education session among HCWs [66]. It was also reported that vaccine hesitancy was improved overtime among HCWs in Singapore [70]. The vaccine acceptance was increased from 42.8% in December 2020 to 76.1% in March 2021 [70]. By November 2021, more than 99.5% of HCWs in the largest healthcare cluster in Singapore had been fully vaccinated [31].

### Limitations

Our study has some limitations. Firstly, we do not have data on the duration of quarantine which may affect QoL differently. However, the quarantine policy was changed only 2 months before our study (the quarantine period was 14 days before 11 Sept 2021 and 10 days after [71]], and hence, it is likely that almost all quarantined participants in our study had been subjected to the same quarantine period. We suggested that future studies should take into account of quarantine duration and places (dedicated facility or home quarantine) to inform strategies for future pandemics. Secondly, as this is a cross-sectional study, we cannot assess causation and the findings may also be biased by the timing of the study. When we conducted this study, the daily case number of COVID-19 positive patients was low (below 100) compared to the highest number in April 2020 (> 800) [1] and there was a high hope of returning to normal with the availability of vaccines. This hope was dampened with the emergence of delta and omicron variants and the surge in positive COVID-19 cases where daily cases were in a few thousands which was substantially higher than that of study period. Hence, the findings may not be applicable to current situation. Longitudinal studies are warranted to understand the changes in QoL in different waves of the pandemic. Lastly, the survey response rate was only 34% and hence, the generalizability of the findings may be limited. However, our study includes both medical and non-medical staff and the survey was opened for six weeks to reduce the low response rate due to the limited time frame. In addition, our study is one of the first few studies which comprehensively assess the impact of the pandemic on QoL of HCWs.

## Conclusions

In conclusion, our study identified high-risk groups and protective factors for QoL at individual and organizational levels. Intervention strategies aiming to improve the QoL of HCWs should prioritize these high-risk groups using a multifaceted multilevel—approach.
